# Dermal papilla cells and melanocytes response to physiological oxygen levels depends on their interactions

**DOI:** 10.1111/cpr.13013

**Published:** 2021-06-08

**Authors:** Carla M. Abreu, Rui L. Reis, Alexandra P. Marques

**Affiliations:** ^1^ 3B's Research Group, I3Bs ‐ Research Institute on Biomaterials, Biodegradables and Biomimetics University of Minho, Headquarters of the European Institute of Excellence on Tissue Engineering and Regenerative Medicine, AvePark, Parque de Ciência e Tecnologia, Zona Industrial da Gandra Guimarães Portugal; ^2^ ICVS/3B’s – PT Government Associate Laboratory Guimarães Portugal

**Keywords:** cellular interactions, dermal papilla cells, hair follicle, melanocytes, physoxia

## Abstract

**Background:**

Human dermal papilla (DP) cells and melanocytes (hMel) are central players in hair growth and pigmentation, respectively. In hair follicles (HFs), oxygen (O_2_) levels average 5%, being coupled with the production of reactive oxygen species (ROS), necessary to promote hair growth.

**Materials and Methods:**

DP cell and hMel proliferation and phenotype were studied under physiological (5%O_2_, physoxia) or atmospheric (21%O_2_, normoxia) oxygen levels. hMel‐DP cells interactions were studied in indirect co‐culture or by directly co‐culturing hMel with DP spheroids, to test whether their interaction affected the response to physoxia.

**Results:**

Physoxia decreased DP cell senescence and improved their secretome and phenotype, as well as hMel proliferation, migration, and tyrosinase activity. In indirect co‐cultures, physoxia affected DP cells’ alkaline phosphatase (ALP) activity but their signalling did not influence hMel proliferation or tyrosinase activity. Additionally, ROS production was higher than in monocultures but a direct correlation between ROS generation and ALP activity in DP cells was not observed. In the 3D aggregates, where hMel are organized around the DP, both hMel tyrosinase and DP cells ALP activities, their main functional indicators, plus ROS production were higher in physoxia than normoxia.

**Conclusions:**

Overall, we showed that the response to physoxia differs according to hMel‐DP cells interactions and that the microenvironment recreated when in direct contact favours their functions, which can be relevant for hair regeneration purposes.

## Graphical abstract

Under physiological oxygen levels (5% O_2_, physoxia), both the highly specialized human dermal papilla (DP) cells and melanocytes (hMel) show increased proliferative capacity and functionality than under atmospheric oxygen levels (21% O_2_). Interestingly, DP cells and hMel response to physoxia varied not only if in co‐culture, but also whether they were interacting indirectly or directly. When hMel and DP cells were directly contacting in organized 3D cell aggregates that better resembled the HF microarchitecture and respective phenotype, the microenvironment recreated under physoxia better favoured their highly specialized functions.

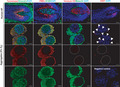

## 1 INTRODUCTION

Hair growth is mainly controlled by the dermal papilla (DP), the hair follicle (HF) inductive mesenchymal structure, whereas its pigmentation relies on the melanogenic activity of follicular melanocytes.^1^, ^2^ These melanocytes represent the progeny of melanoblasts residing in the bulge, which proliferate and migrate to the hair bulb, surrounding the DP and starting to produce and transfer melanin to the keratinocytes of the growing shaft.^3^, ^4^, ^5^, ^6^, ^7^ Although the DP is considered the HF control centre, and its anatomical proximity with bulbar melanocytes implies a role also in hair pigmentation, little is known about the capacity of DP cells to regulate melanocytes. Rodent studies indicate that DP cells can influence melanocytes proliferation/differentiation, migration and affect pigment formation and hair coat colour.^8^ In vitro studies confirmed a chemotactic effect of DP cell‐conditioned medium towards human melanocytes (hMel),^5^ further suggesting mediation of melanocytes location and migration in the HF by DP cells. Interestingly, DP cells’ extracellular matrix (ECM) was also suggested to stimulate tyrosinase activity,^9^, ^10^ the rate‐limiting step for melanin production.^11^

Oxygen (O_2_) is a basic component of the tissue's microenvironment, and their fluctuation can deeply affect cellular metabolism, signalling, proliferation, differentiation and reactive oxygen species (ROS) formation.^12^, ^13^ Physiological ROS play a regulatory role in several cellular signalling events.^14^, ^15^, ^16^ For example, melanogenesis itself is a ROS generator cellular process, but melanocytes have mechanisms to cope with oxidative stress and avoid cellular damage. These include, among others, upregulation of the antioxidant response,^17^, ^18^ and expression of the nuclear erythroid 2‐related factor (NRF2)^19^, ^20^ or the Ataxia Telangiectasia Mutated (ATM) protein.^21^ In opposition, uncontrolled levels of ROS have been linked to the aetiopathogenesis of several conditions, including androgenetic alopecia and hair greying.^22^, ^23^ It is well known that ROS accumulate at supraphysiological oxygen levels.^24^ Previous studies demonstrated that in cultures performed under 21% O_2_ (normoxia) both HF mesenchymal (DP and dermal sheath cells)^25^ and epithelial^26^ populations proliferate at lower rates than when respectively cultured at 6% O_2_ or 4% O_2_. Moreover, under hypoxic conditions (2% O_2_), DP cells viability, phenotype and inductivity are improved.^23^, ^27^ Further, hMel proliferation and melanin production are also higher at 1%‐5% O_2_ than above normoxia.^28^ Overall, these studies indicate that low oxygen levels are beneficial for DP cells and melanocytes, which seem to agree with the oxygen tension measured in the human DP (4.0%‐5.2% O_2_)^29^ or skin (average 5.3% O_2_).^30^ Nevertheless, the anagen hair bulb is a ROS‐enriched microenvironment,^31^ in which ROS directly activates proliferation and differentiation programs, stimulating hair growth.^32^ Therefore, despite the involvement of oxygen‐associated responses, potentially by the different cells implicated in hair growth is expected, it remains to be elucidated.

Considering this, we investigated the response of hMel and DP cells to physiological oxygen levels (5% O_2_, physoxia) and if this response was influenced by their interaction, aiming at mirroring their potential communication within the HF. We demonstrate that under physiological culture conditions, together with an expected decrease in ROS levels, both DP cells and hMel showed increased proliferative capacity and functionality. Interestingly, DP cells and hMel response to physoxia varied not only if these were co‐cultured, but also whether they were indirectly or directly interacting. When hMel and DP cells were directly contacting in 3D cell aggregates resembling their native organization, the microenvironment recreated under physoxia favoured their highly specialized functions.

## 2 METHODS

### 2.1 Cell culture

DP cells were isolated^33^ from HF occipital scalp samples obtained from consenting patients who underwent hair transplantation procedures. DP cells were sub‐cultured on bovine collagen‐coated (Sigma‐Aldrich) surfaces in Dulbecco's modified eagle's medium (DMEM, Sigma‐Aldrich) with 10% foetal bovine serum (FBS) and 1% antibiotic‐antimycotic solution (Gibco). Neonatal foreskin hMel were purchased from Cell Applications (catalog no. 104‐05n) and cultured in the recommended HEM complete medium. Unless otherwise stated, the cell densities used to establish the monocultures were 2x10^4^ cells/cm^2^ (DP cells and hMel).

Physoxia cultures were established with 5% O_2_ in a hypoxic chamber (Coy O2 Control Glove Box; Coy Laboratory Products). Cells cultured under normoxia were used as controls. Cells were used up to passage 4 (hMel) or passage 7 (DP cells).

To assess the link between ROS and ALP, DP cells were cultured overnight and then treated with hydrogen peroxide (H_2_O_2_; 300 μΜ, PanReac AppliChem), with *N*‐acetyl cysteine (NAC; 1 mM, Sigma‐Aldrich) or with NAC for 2h before H_2_O_2_.

### 2.2 Morphology and aggregation analysis

After 3 days of culture, DP cells were fixed with 10%‐formalin for 15 minutes at room temperature (RT) and their F‐actin filaments stained with Phalloidin‐TRITC (0.1 mg/mL, Sigma‐Aldrich) for 1 hour at RT. Nuclei were counterstained with 4,6‐diamidino‐2‐phenylindole (DAPI) (1:1000, Biotium) for 15 minutes at RT. Images (six per triplicate) were acquired with an Axio Observer microscope (Zeiss) and used for the quantification of cell area, perimeter and major axis length, with the software module “*MeasureObjectSizeShape*” of CellProfiler^TM^ 3.0.0.^34^

Cell aggregation was analysed after 7 days of culture, after labelling the cells' nuclei with DAPI. Images (ten per triplicate) were acquired (Axio Observer, Zeiss) and analysed with the CellProfiler 3.0.0^TM^ module “*RelabeledNuclei*”. Cells were considered adjacent if the distance between their nuclei was below 8 pixels. Groups of 30 or more adjacent cells were counted as one aggregate.

### 2.3 Senescence‐associated‐β‐Galactosidase assay

DP cells were seeded at a density of 1 x 10^4^ cells/cm^2^ and cultured overnight. Next day, the cells medium was replaced by serum‐free DMEM and DP cells were kept in culture for 5 days. The detection of senescence cells was performed using the Senescence β‐Galactosidase Staining Kit (Cell Signaling Technology) following manufacturer instructions. Images (12 per each triplicate) used to quantify the percentage of senescent cells were taken using an AxioVert.A1 microscope (Zeiss).

### 2.4 Quantification of collagenous and non‐collagenous proteins

The total amount of collagenous (COL) and non‐collagenous (NCOL) proteins were quantified using the Sirius Red/Fast Green Staining Kit (Chondrex) according to the supplier instructions. DNA values were used to normalize data.

### 2.5 Migration assay

hMel (4 x 10^4^ cells/cm^2^) were seeded in 8.0 µm pore size inserts (Corning) in non‐supplemented HEM medium, while complete medium was added to the bottom well. After 48 hours of culture under normoxia or physoxia, cells that migrated from the insert to the bottom well were fixed with 10% formalin (15 minutes, RT). Images were acquired with an AxioVert.A1 (Zeiss) for quantification (12 per triplicate) or with an Axio Observer (Zeiss) microscope after staining the cells' nuclei with DAPI.

### 2.6 Tyrosinase activity quantification

hMel were incubated for 5 minutes (ice) with 20 mmol/L Tris (hydroxyethyl) aminomethane (pH 7.5) containing 0.1% Triton X‐100 and a protease inhibitor cocktail (1:100, Sigma‐Aldrich). Cell lysates were then centrifuged at 14 500 rcf (10 minutes, 4°C) and 70 μL of the supernatant transferred into transparent 96‐well plates. As a substrate for tyrosinase, a 0.1% (wt/v) L‐Dopa (Sigma‐Aldrich) solution was prepared in sodium phosphate buffer (pH 6.8) and 140 μL were added to each well. Plates were incubated for 2 hours at 37°C, and the absorbance measured at 475 nm using a microplate reader (Synergy HT, BioTek). Data are presented as relative tyrosinase activity after normalization with DNA values.

### 2.7 Co‐cultures

DP cells resuspended in DMEM with 10% FBS were seeded at a density of 2 x 10^4^ cell/cm^2^ and cultured overnight before establishing the co‐culture with hMel, seeded at 2 x 10^4^ cell/cm^2^ in HEM in 0.4 µm pore size inserts (Corning). Monocultures of each cell type were prepared as controls, either by culturing cells in their regular medium (DMEM or HEM), or in the medium used in to establish the co‐culture, DMEM with HEM at a 1:1 ratio (DMEM:HEM), to control possible medium effects. Direct co‐cultures were established by seeding 3 x 10^3^ DP cells in round bottom ultra‐low attachment 96 wells (Corning) in 50 µL of DMEM with 10% FBS for 24 hours before the addition of 1.5 x 10^3^ hMel resuspended in 25 µL of HEM medium. Both co‐cultures were performed for 3 days.

### 2.8 DNA, active alkaline phosphatase and ROS quantification

Cells were lysed in water with 0.01% sodium dodecyl sulphate. A 1 hour incubation at 37°C was followed by freezing at −80°C. DNA content was quantified using the Quant‐iT™ PicoGreen® dsDNA kit (Thermo Fisher Scientific), and ROS levels were measured using the OxiSelect™ In vitro ROS/RNS assay kit (Cell Biolabs Inc). Cell lysates were also used to quantity active ALP levels in DP cells, using the Alkaline Phosphatase Detection Kit (Sigma‐Aldrich). For DNA and ALP quantification in cell aggregates, a 5s sonication step (ice) was first performed to ensure the complete disintegration of the 3D aggregates prior quantification. All commercial kits were used according to the manufacturer instructions. DNA values were used to normalize ROS and ALP results.

### 2.9 Alkaline phosphatase staining

The detection of active ALP was performed by incubating formalin‐fixed DP cells (15 minutes, RT) for 20 minutes with a solution prepared with 5 μL of *p*‐nitroblue tetrazolium chloride (NBT) and 3.75 μL of 5‐bromo‐4‐chloro‐3‐indolyl phosphate (BCIP, Roche) in 1 mL of staining buffer [100 mmol/L NaCl, 100 mmol/L Tris‐HCl (pH 9.5) and 50 mmol/L MgCl_2_ in water]. Representative images were acquired with an AxioVert.A1 microscope (Zeiss).

### 2.10 ELISA

After co‐culture, inserts containing the hMel were removed and DP cells were cultured in serum‐free DMEM for 24 hours. The supernatant of the cells was then collected, centrifuged (1000 *g*, 10 minutes) and single‐use aliquots were stored at −80°C. Human vascular endothelial growth factor (VEGF) ELISA Development Kit (PeproTech) and human bone morphogenetic protein‐2 (BMP2) Standard ELISA Development Kit (Petrotech) were then used following the manufacturer instructions to determine VEGF and BMP2 levels. DNA values were used to normalize data.

### 2.11 Immunofluorescence staining

DP‐hMel aggregates were fixed in 10%‐formalin (overnight, 4°C), embedded in HistoGel^TM^ (Thermo Scientific) and processed for paraffin inclusion. 4‐µm paraffin‐embedded sections were then dewaxed and heat‐mediated antigen retrieval was performed with sodium citrate buffer (pH 6.0). Primary antibodies (Table S1) were detected with Alexa Fluor®488/594 (1:500, Molecular Probes) secondary antibodies and nuclear counterstain was performed with DAPI. Images were acquired using an Axio Imager Z1m microscope (Zeiss).

Haematoxylin and eosin (H&E) staining was performed according to standard protocols and representative images acquired with a DM750 microscope (Leica). Images were used to count cell nuclei and determine the DP cells/hMel ratio present within the cell aggregates for normalization of tyrosinase and ALP activity using the DNA amount of the corresponding cells.

### 2.12 Statistical analysis

Statistical analysis and data visualization were performed using the GraphPad Prism 7.03. The D’Agostino & Pearson normality test was used to determine whether data followed a Gaussian distribution. Non‐parametric data were analysed with a two‐tailed Mann‐Whitney test (two groups, unpaired) or with a Friedman (paired) or Kruskal‐Wallis test (unpaired) when more than 2 groups were compared. Parametric data were analysed with a two‐tailed Student's *t* test (two groups, paired or unpaired). The comparison of more than two groups was performed with an ordinary (unpaired) or RM (paired) one‐way ANOVA (one independent variable) or two‐way ANOVA (two independent variables). Data are presented as mean ± standard error of the mean (SEM). For data displayed as dot plots, black dots represent data points and red bars indicate the mean. Differences with *p*‐values <.05 were considered significant.

## 3 RESULTS

### 3.1 Physoxia reduces the negative impact of in vitro culture conditions on DP cells

**FIGURE 1:**
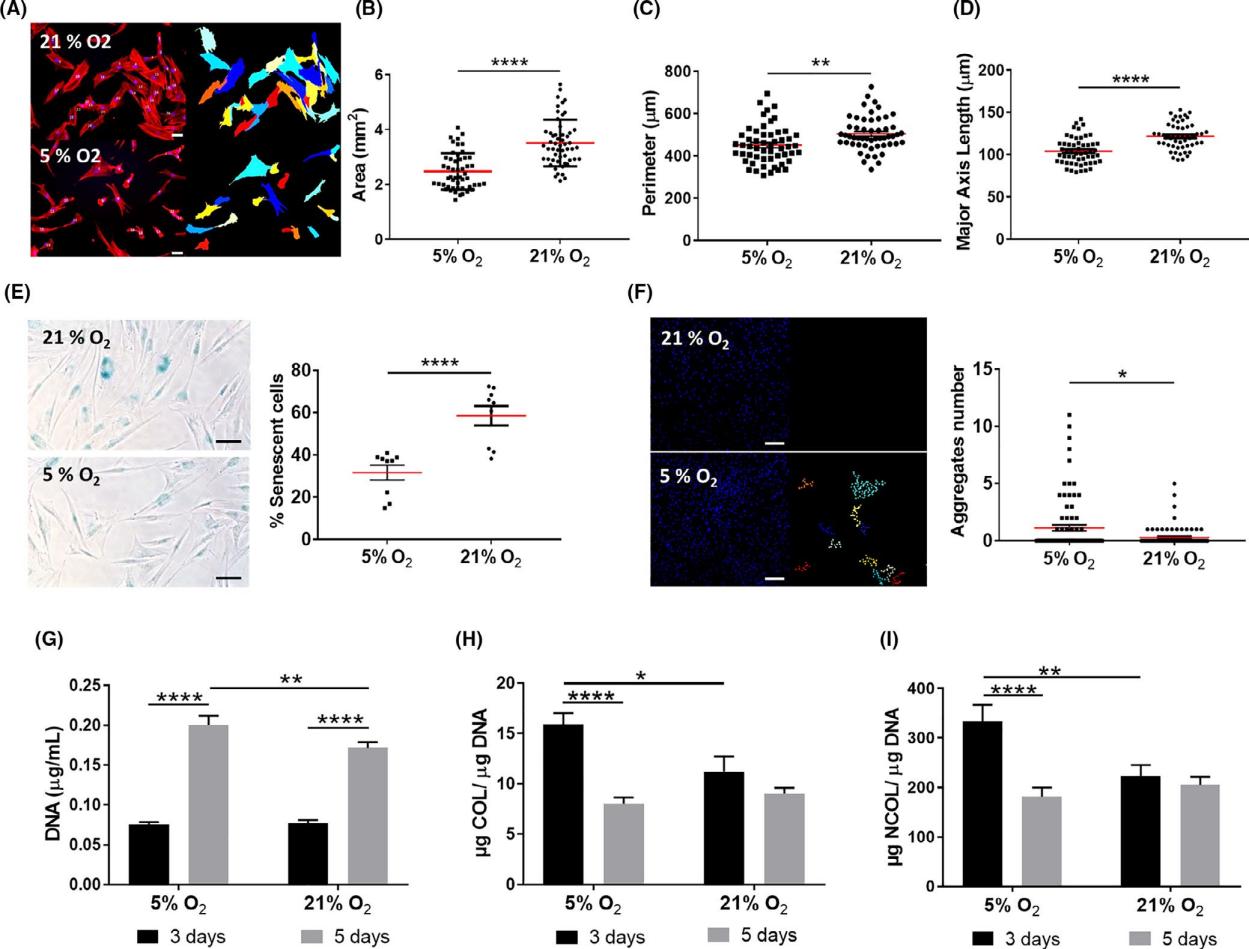
DP cells phenotype under physoxia. (A) Representative images of DP cells F‐actin cytoskeleton labelled with Phaloidin‐TRITC (left panel) and respective CellProfiler^TM^ output (right panel) used to quantify morphological features such as (B) cell area, (C) perimeter and (D) major axis length. Nuclei were counterstained with DAPI. Significant differences between DP cells cultured under normoxia (21% O_2_) or physoxia (5% O_2_) were analysed using an unpaired, two‐tailed Student's *t* test (n = 3). Scale bar = 50 µm. (E) Representative images of the β‐galactosidase staining used to quantify the percentage of senescent DP cells. Significant differences were analysed using a paired, two‐tailed Student's *t* test (n = 3). Scale bar = 50 µm. (F) Representative images of DAPI‐labelled DP cells (left panel) and CellProfiler^TM^ output of subsequent grouping of related nuclei (distance < 8 pixels) used to count the number of cell aggregates (≥30 related nuclei). Significant differences were analysed using an unpaired, two‐tailed Mann‐Whitney test (n = 3). Scale bar = 200 µm. (G) DNA quantification used to evaluate cellular proliferation. Differences among oxygen levels at the same time point and differences for the same oxygen level along time were analysed using an unpaired, two‐tailed Mann‐Whitney test (n = 3). Quantification of (h) COL and (i) NCOL proteins secretion. Results were analysed using an unpaired, two‐way ANOVA. All data are presented as mean ± SEM, and statistical differences are indicated as *P <.05; **P <.01; ****P <.0001

DP cell cultures are typically established under normoxia, rapidly losing their native phenotype and intrinsic properties,^35^, ^36^ including their key self‐aggregation capacity.^37^, ^38^ Moreover, they also have a short lifespan ^39^ in culture, which is accompanied by morphological changes such as shifting from a small polygonal morphology to a spindle‐like shape,^40^, ^41^ before acquiring an enlarged morphology.^23^ Therefore, we investigated whether those changes also occurred under physoxia to understand how the O_2_ level impacts DP cells’ phenotype in culture. DP cells under physoxia depicted a polygonal and less spindle‐like shape and higher nuclei‐to‐cytoplasm ratio, as demonstrated by the significant decrease in the cells’ area, perimeter and major axis length in comparison with normoxia (Figure 1A‐D). Physoxia also significantly decreased the percentage of senescent DP cells in culture (Figure 1E) and improved their aggregative capacity (Figure 1F). Moreover, it enhanced cell proliferation, albeit the DNA amount at day 3 was similar in normoxia and physoxia (Figure 1G). Interestingly, an opposite effect was observed regarding COL (Figure 1H) and NCOL (Figure 1I) protein secretion under physoxia, which was only beneficial after 3 days in culture. Altogether, these results suggest that physoxia promotes a healthier state in cultured DP cells, which featured characteristics typically associated with low passage cells.

### 3.2 Physoxia enhances hMel migration, tyrosinase activity and proliferation within short culture times

**FIGURE 2:**
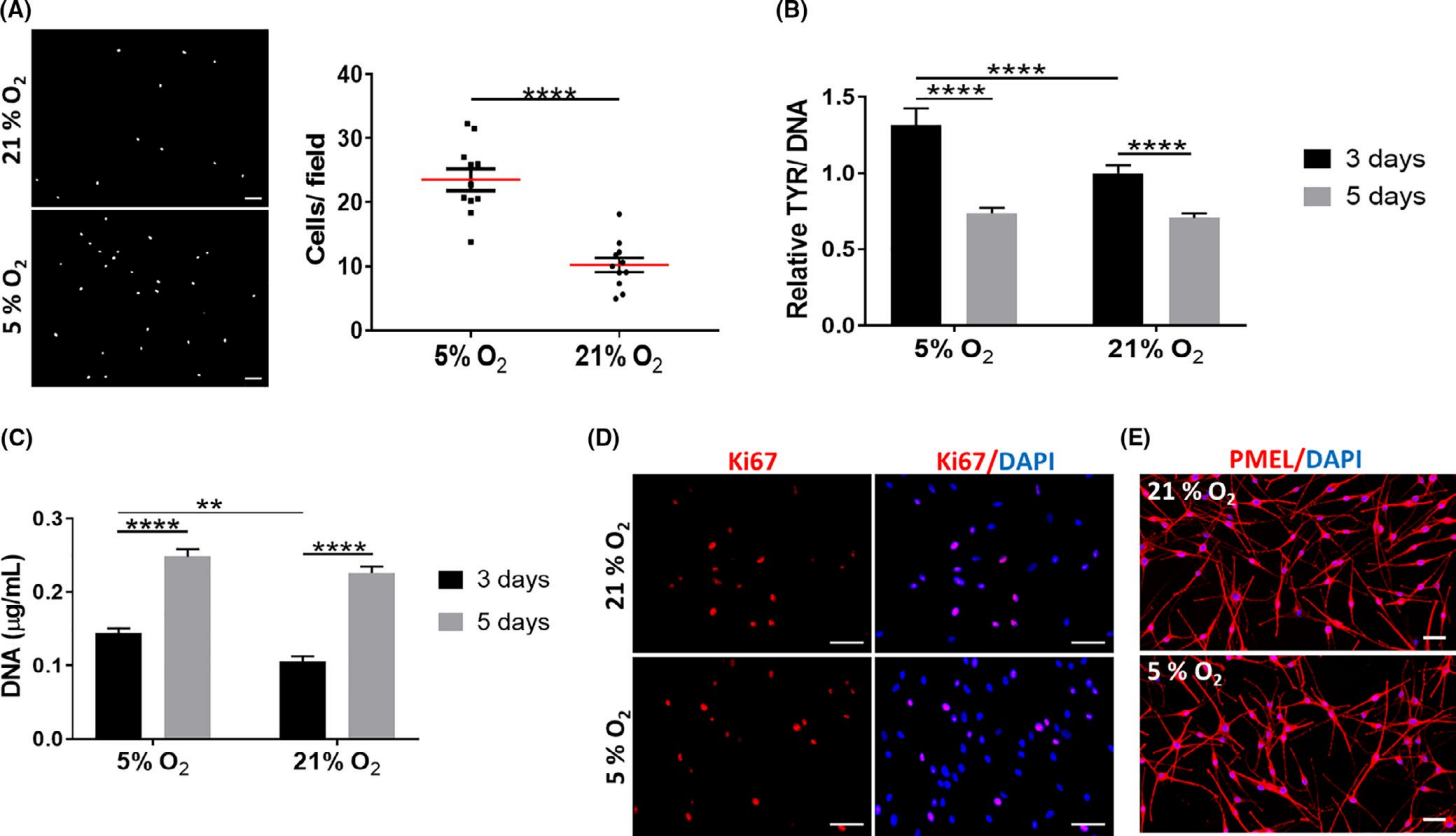
Characterization of hMel behaviour under physoxia. (A) Representative fluorescence microscopy images of DAPI labelled hMel that migrated over 48 h and respective quantification. Scale bar = 100 µm. (B) Quantification of hMel tyrosinase activity (TYR). Relative values in comparison with cells cultured under 21% O_2_ at day 3 of culture are presented. Significant differences were determined by a paired, two‐way ANOVA (n = 3). (C) DNA quantification used to assess hMel cellular proliferation. Significant differences were analysed using an unpaired, two‐way ANOVA (n = 3). (D) Representative immunofluorescence images showing Ki67‐positive hMel. Nuclei were counterstained with DAPI. Scale bar = 50 µm. (E) PMEL immunostaining showing hMel morphology. Nuclei were counterstained with DAPI. Scale bar = 50 µm. Significant differences were analysed using a paired, two‐tailed Student's *t* test (n = 3). All data are presented as mean ± SEM, and statistical differences are indicated as **P < .01; ****P < .0001

Although hMel are normally cultured under normoxia, there are indications that their proliferation and tyrosinase activity are favoured under lower oxygen tensions.^28^ We found that physoxia significantly increased both hMel migration (Figure 2A) and tyrosinase activity (Figure 2B), although this last effect was not sustained along with the culture. Similarly, significantly higher DNA levels were observed for hMel cultured under physoxia at day 3 of culture (Figure 2C), suggesting an improved proliferative capacity. This effect was lost with the culture time, despite the high number of Ki67‐positive cells (Figure 2D). Physoxia did not seem to affect the morphology of hMel (Figure 2E). Collectively, these results indicate that physoxia supports hMel functional features better than normoxia but only for short culture periods.

### 3.3 DP cell and hMel response to physoxia depends on their type of interaction

**FIGURE 3:**
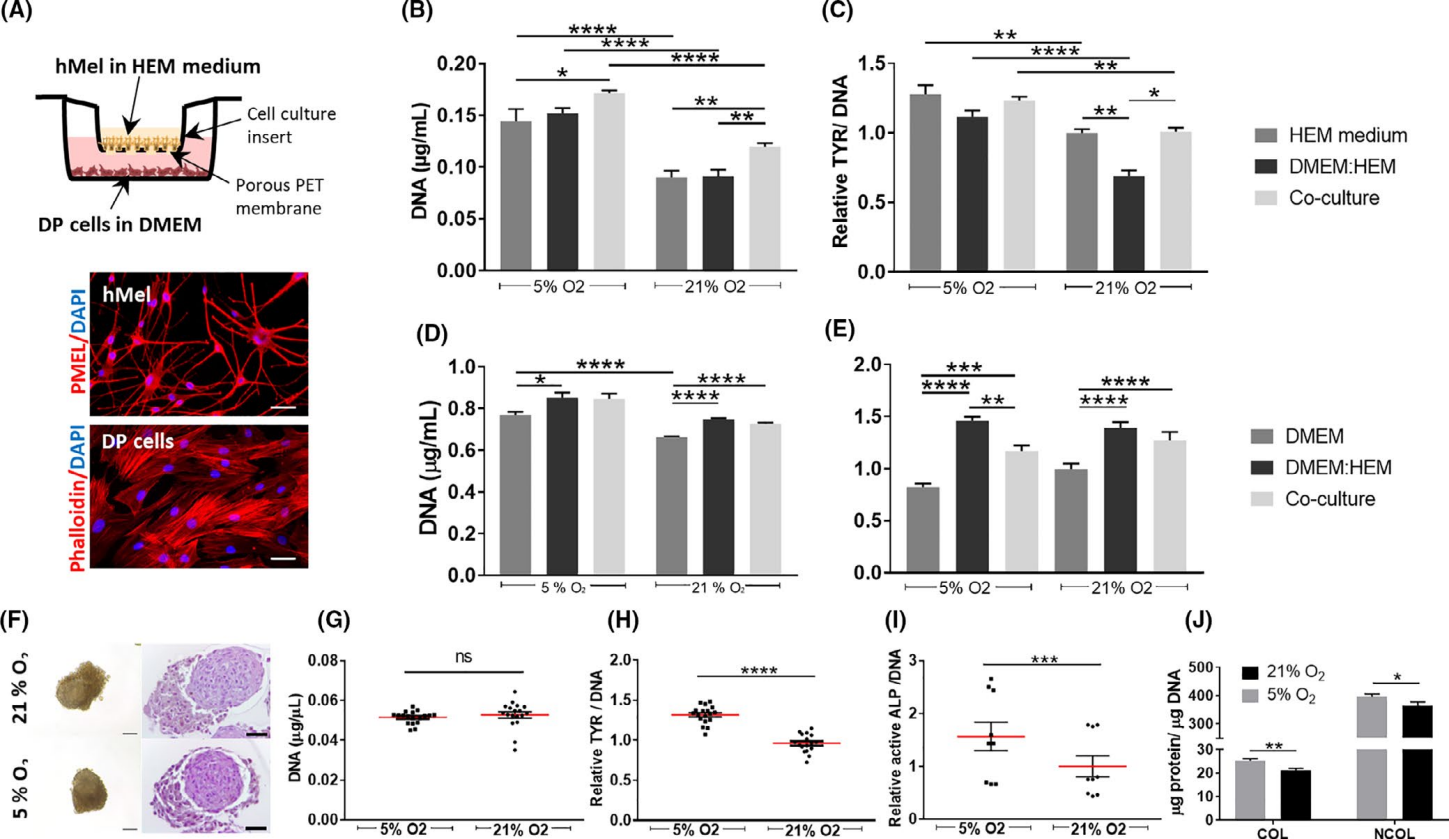
Physoxia effects on co‐cultured hMel and DP cells. (A) Schematic representation of the indirect co‐culture system used to study hMel and DP cells interactions, accompanied by images displaying the morphology of each cell type, respectively, immunolabelled with PMEL and stained with Phalloidin‐TRITC. Nuclei were counterstained with DAPI. Scale bar = 50 µm. (B) DNA quantification used to assess hMel cell numbers in the co‐culture with DP cells, and in the respective homotypic controls—hMel cultured in standard conditions (HEM medium) or in the medium used for the co‐culture (DMEM:HEM). Data were analysed using an unpaired, one‐way ANOVA (n = 3). (C) Quantification of TYR in hMel in the different culture conditions. Statistical differences were calculated using an unpaired, Kruskal‐Wallis test (n = 3). Quantification of (D) DNA and (E) active ALP of DP cells co‐cultured with hMel and in the respective control media. Significant differences were analysed using a paired, Friedman test (n = 6 for DNA; n = 3 for active ALP). (F) Representative phase contrast (left panel) and H&E (right panel) images of the cell aggregates formed after direct culture of hMel with DP spheroids. Scale bars are 100 µm and 50 µm, respectively. (G) Quantification of DNA of the aggregates. *P* values were calculated using an unpaired, two‐tailed Mann‐Whitney test (n = 3). (H) Quantification of TYR in hMel in the aggregates. Differences were calculated using a paired, two‐tailed Student's *t* test (n = 3). (I) Quantification of active ALP in the DP spheroids. A paired two‐tailed Student's *t* test was used to perform the statistical analysis (n = 3). (J) Quantification of COL and NCOL proteins present in the aggregates. Statistical analysis was performed using a paired Wilcoxon signed‐rank test (COL) or a paired two‐tailed Student's *t* test (NCOL) (n = 4). Relative values are presented in comparison with the cells standard culture conditions at 21% O_2_ (C,D) or in comparison with aggregates formed at 21% O_2_ (H,I). All data are presented as mean ± SEM and statistical differences are indicated as *P <.05; **P <.01; ***P <.001; ****P <.0001

Although residing in close vicinity in the hair bulb and having their functions coupled to anagen,^42^, ^43^ little is known about how human DP cells and hMel interact and potentially affect each other's functionality. Knowing that physoxia individually improved hMel and DP functional features after 3 days in culture, we then explored its effect when these cells were indirectly co‐cultured (Figure 3A). The co‐culture with DP cells did not add to the increased hMel proliferation induced by physoxia (Figure 3B), in opposition to normoxia. Like for proliferation, co‐culture with DP cells under physoxia did not affect tyrosinase activity in hMel, contrarily to normoxia, which promoted a recovery from the negative effect of the co‐culture medium (Figure 3C).

Regarding DP cells, they proliferated significantly more under physoxia than in normoxia but only when cultured in their conventional medium. Thus, the higher DNA amount detected in co‐culture might be due to the medium used. This is also sustained by the results similar to the control condition, both under physoxia and normoxia (Figure 3D). Active ALP levels in co‐cultured DP cells were not affected by physoxia but, as for proliferation, the medium used led to a significant increase of this inductive marker. In the presence of hMel, a significant decrease of DP cells’ active ALP was observed under physoxia, but not in normoxia (Figure 3E).

In the HF, hMel and DP cells are separated only by a thin and permeable basal lamina.^2^ Therefore, we sought to investigate whether physoxia effects were different from those observed in the indirect co‐cultures, assuming a direct interaction between hMel and DP cells. When hMel were cultured with DP spheroids, they organized themselves around the spheroid, displaying a polarized position over one‐half of the DP spheroid, independently of the oxygen level (Figure 3F). Highly stable aggregates with similar DNA content were obtained (Figure 3G). Interestingly, the phenotype of both cell types was improved under physoxia, as demonstrated by a significant increase of tyrosinase activity in hMel (Figure 3H) and by the higher amount of active ALP in DP cells (Figure 3I), their main functional markers, respectively. Moreover, the production of COL and NCOL proteins by aggregates cultured in physoxia was significantly higher than in normoxia (Figure 3J). Physoxia benefits hMel and DP cells functionality when both cell types are directly contacting. Interestingly, hMel response to physoxia does not seem to be indirectly affected by DP cells, while hMel signalling appears to have an impact on DP cells’ ALP activity.

### 3.4 ROS generation due to hMel and DP cells interaction does not directly correlate with DP cells functionality

**FIGURE 4:**
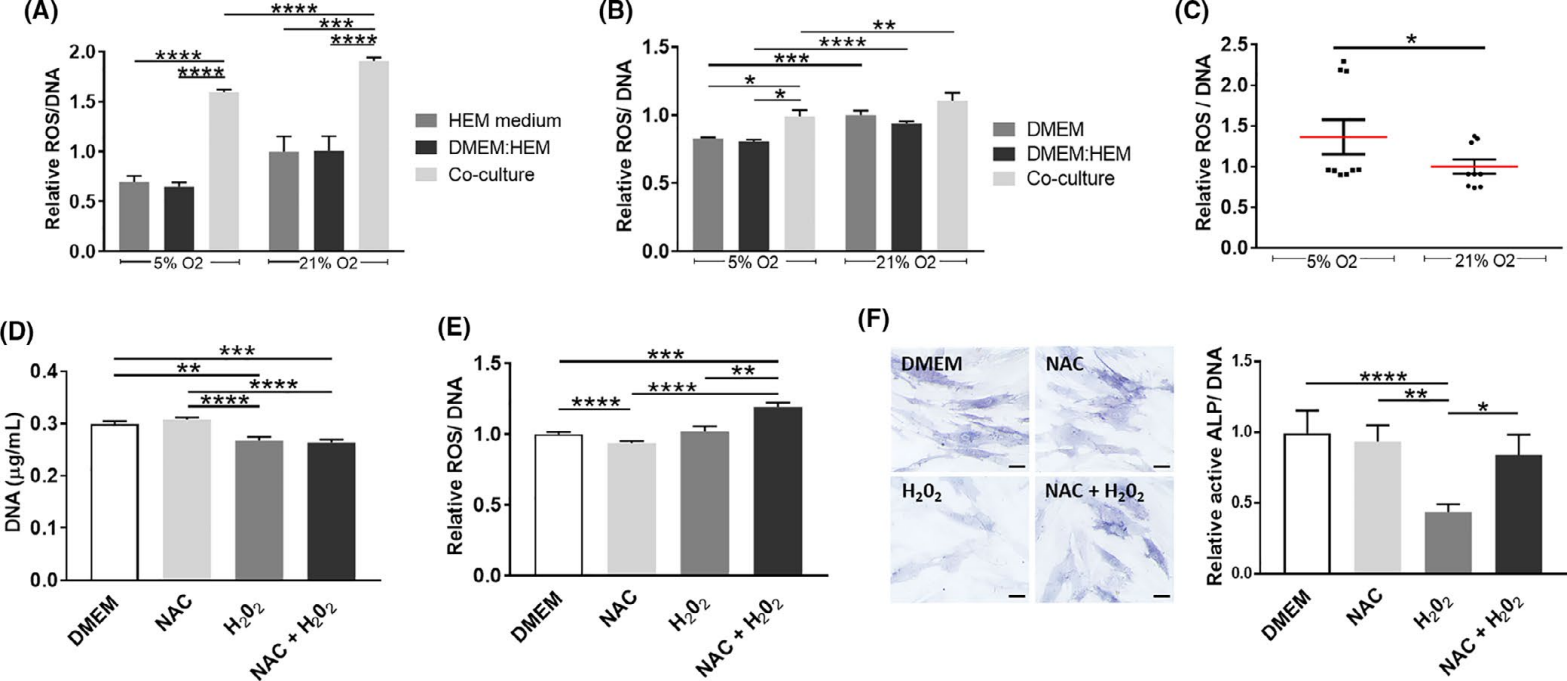
ROS production by co‐cultured hMel and DP cells and analysis of its association to active ALP in DP cells. ROS quantification in (A) hMel and (B) DP cells co‐cultured indirectly and in the respective control conditions. Significant differences were analysed using an unpaired, one‐way ANOVA (n = 3). (C) Amount of ROS in the DP‐hMel cell aggregates. Statistical analysis was performed using a paired, two‐tailed Student's *t* test (n = 3). (D) Quantification of the DNA amount in conventionally cultured DP cells (DMEM, 21% O_2_) in the presence of the ROS‐scavenger NAC, H_2_O_2_ or the combination of both (NAC + H_2_O_2_). A paired Friedman test was used to identify significant differences (n = 3). (E) Quantification of ROS production by DP cells after NAC, H_2_O_2_ or both treatments. Statistical analysis was performed using a paired, one‐way ANOVA (n = 3). (f) Representative light microscopy images of ALP staining in DP cells and respective quantification of active ALP levels. Significant differences were calculated using a paired, Friedman test (n = 3). Scale bar = 100 µm. Relative values are presented in comparison with the cells standard culture conditions at 21% O_2_ (A,B,E,F) or in comparison with aggregates formed at 21% O_2_ (C). All data is presented as mean ± SEM and statistical differences are indicated as *P < .05; **P < .01; ***P < .001; ****P < .0001

During hair growth and pigmentation, the bulb is a ROS‐enriched environment^31^; therefore in addition to the functionality of hMel and DP cells, we addressed the involvement of ROS in their response. The production of ROS by hMel was significantly lower under physoxia, although this effect was significant only for the co‐cultures (Figure 4A). Moreover, hMel in co‐culture produced significantly more ROS than in the control conditions, regardless of the oxygen level. Physoxia also led to a reduction of ROS levels in DP cells in comparison with normoxia, independent of the culture conditions (Figure 4B). The indirect co‐culture with hMel under physoxia also resulted in significantly higher amounts of ROS than in control conditions (Figure 4B). Surprisingly, when cells were directly cultured, ROS production in physoxia was significantly higher than in normoxia (Figure 4C), the opposite of what was observed in indirect co‐cultures.

Considering that in indirect (Figures 3E and 4B) or direct (Figures 3I and 4C) co‐cultures the effect of physoxia over the amount of active ALP and ROS followed a common trend, we then investigated whether there was a correlation between these responses. For that, DP cells were treated with H_2_O_2_ (exogenous ROS) to increased oxidative stress, with the ROS inhibitor NAC or both. Treatment with H_2_O_2_ led to a significant decrease in the amount of DNA (Figure 4D) but surprisingly, it did not affect ROS intracellular levels in DP cells (Figure 4E) while a significant decrease of active ALP was observed (Figure 4F). Moreover, NAC pre‐treatment before H_2_O_2_ addition further enhanced ROS production in comparison with H_2_O_2_ alone, but it also reduced the H_2_O_2_ effect on the amount of active ALP. Although it is not clear the mechanism by which H_2_O_2_ decreases ALP activity in DP cells, these results suggest that it is not directly correlated with an increase in ROS levels.

### 3.5 Physoxia and 3D co‐culture supports DP cells and hMel phenotype

**FIGURE 5:**
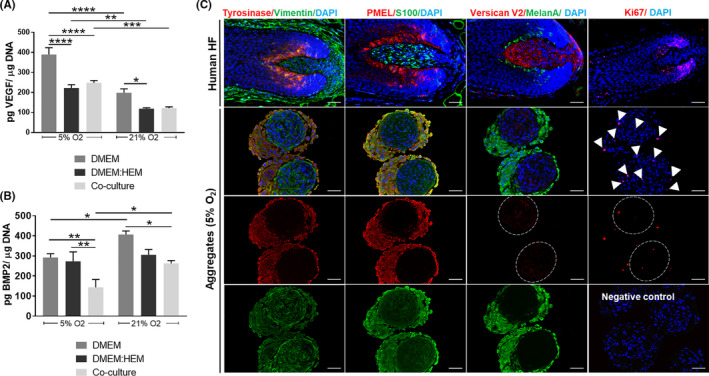
Phenotypic assessment of co‐cultured DP cells and hMel. ELISA quantification of (A) VEGF and (B) BMP2 released by DP cells indirectly co‐cultured with hMel and the respective control conditions. Results are shown as mean ± SEM. Significant differences were analysed using an unpaired, one‐way ANOVA (n = 3) and are indicated as *P < .05; **P < .01; ***P < .001; ****P < .0001. (C) Representative immunofluorescence images showing the expression of different DP cells and hMel markers in the native hair follicle (HF) tissue and in the DP‐HMel aggregates prepared under physoxia. Nuclei were counterstained with DAPI. White arrowheads indicate Ki67‐positive cells and dotted circles delimitate the DP spheroid area. Scale bars = 50 μm

To further explore both physoxia and hMel influence on DP cells hair regenerative potential, we looked at the production of a known promoter^44^, ^45^ or inhibitor factor^46^ of hair induction, namely VEGF and BMP2, respectively. In physoxia, the amount of VEGF released by DP cells was significantly higher than in normoxia independently of the culture condition (Figure 5A). The co‐culture medium negatively impacted VEGF release by DP cells, which was not overcome with the co‐culture with hMel. In opposition, the amount of BMP2 in physoxia was significantly lower than in normoxia, for both co‐cultures and conventional DP culture medium (Figure 5B). As this effect was not seen in the control established with the co‐culture medium, it suggests that hMel presence was essential for the observed result.

Considering that hMel‐DP cell aggregates better resemble the HF bulb than plain 2D cultures, and the observed advantages regarding their functionality under physoxia, we then investigated the rescue of cell's native‐like phenotype under these conditions. The expression profile of different DP and hMel markers in the 3D aggregates (Figure 5C) showed that the cells display a phenotype similar to the native HF (Figure 5C, upper panel). The identification of specific markers of hMel—tyrosinase, PMEL, MelanA—and markers expressed also by DP cells—vimentin and S100—showed clear compartmentalization between the cell types. Moreover, Ki67 immunolabeling confirmed a low number of proliferative cells within both cellular compartments (Figure 5C). Noteworthy, tyrosinase expression in the cellular aggregates was higher than in conventionally cultured hMel (Figure S1). Moreover, the in vivo predominant V2‐isoform of versican,^47^ a DP inductive marker typically absent in 2D‐cultured cells (Figure S1) was weakly expressed in the cellular aggregates, confirming what was previously described for DP spheroids.^35^

Overall, these results indicate that under physoxia DP cells’ inductive secretome is promoted which, in conjunction with 3D‐culture conditions, allows an improved recovery of hMel and DP cell functional markers.

## 4 DISCUSSION

Besides ensuring overall cellular survival, oxygen levels are responsible for regulating a wide range of tissue functions, the reason why each organ, or even tissue, has its oxygenation status.^12^ In the HF and skin, oxygen ranges about 5% O_2_; however, the anagen hair bulb is a ROS‐enriched microenvironment,^31^ which seems to indicate the involvement of multiple oxygen‐associated responses, potentially by the different cells implicated in hair growth. Therefore, we aimed to explore DP cells and hMel response to physiological oxygen levels, and if this response was influenced by either their indirect signalling or their direct contact, as it happens in the native tissue.

When cultured at standard atmospheric levels, DP cells have a characteristic short lifespan before entering growth arrest^39^ and gradually lose their native properties.^1^ This has been associated with premature senescence in vitro, triggered by excessive ROS production and oxidative stress.^23^ We show that DP cells cultured under physoxia featured an early‐culture morphology and phenotype, characterized by decreased senescence and increased proliferative and aggregative capacity, as previously observed for 2% O_2_ culture conditions.^23^ Moreover, under physoxia ROS levels were lower, which is expected given the reduced oxygen availability for the mitochondrial respiratory chain,^48^ the main toxic ROS producer.^49^ When exposed to excessive ROS, the capacity of DP cells to support hair growth is severely compromised and they lose their hair inductive ability.^50^ Interestingly, DP cells cultured under physoxia released higher amounts of VEGF and reduced levels of BMP2, which corresponds to the necessary trend observed during anagen induction and hair growth initiation,^45^, ^46^, ^51^, ^52^ suggesting that physoxia stimulates DP cells inductive secretome. Further, also in agreement with what referred above to DP cells, hMel cultures under physoxia showed lower ROS levels. This was also associated with an increase of hMel migration, proliferative capacity and tyrosinase activity, confirming the results of a previous study showing higher hMel growth and pigmentation in cultures established under 1%‐5% O_2_.^28^

During the hair growth phase, hMel mature into tyrosinase‐active cells after migrating to the hair bulb and surrounding the DP, and indirect evidence suggests that DP may be involved in this process.^11^ In a previous study, DP cell‐conditioned medium was shown to enhance hMel tyrosinase activity under normoxic conditions.^10^ We show that hMel tyrosinase activity in the presence of DP cells and under physoxia was higher than in normoxia, yet similar to the homotypic controls, suggesting that the influence of DP cell‐produced soluble factors on hMel do not surpass physoxia benefits. Interestingly, in our work hMel tyrosinase activity in the 3D‐aggregates cultured under physoxia also correlated with a higher ECM deposition, which is in agreement with a previous study^10^ that showed improved tyrosinase activity in both hMel directly co‐cultured with DP cells and with their ECM. These seem to support a link between hMel tyrosinase activity and DP cells ECM, in addition to their secretome.

In the HF, DP cells are mitotically quiescent^53^, ^54^ and the differentiated tyrosinase‐active hMel are less committed to cell division.^6^, ^55^ In the indirect co‐cultures, hMel proliferated more in response to physoxia while DP cells remained unaffected, contrarily to the compromised proliferative capacity shown before for both DP cells and hMel cultured under normoxia.^25^, ^28^ Further, the cells’ proliferative capacity in the 3D aggregates was lower than in the indirect co‐cultures that were established in 2D standard conditions, evidencing a mitotic profile that more closely emulates the native behaviour.

Despite the generically accepted deleterious effects of supraphysiological ROS accumulation, there is a thin line separating beneficial and detrimental effects, which is associated to the tissues’ physiological ROS levels. In the hair bulb, a transient and physiological elevation of ROS^31^ is necessary to promote hair growth and differentiation programmes^32^ during anagen. In the indirect co‐cultures, the amount of ROS produced was significantly higher than in the corresponding homotypic controls, independently of the oxygen level, which seems to suggest that cellular crosstalk in positively influencing ROS formation. Intriguingly, ROS levels in the indirect co‐cultures in physoxia were lower than in normoxia, but the opposite effect was observed in the direct co‐cultures (3D aggregates). While the latter seems to contradict the expected link between the available oxygen and ROS formation, it is also known that spheroids represent a hypoxic environment.^56^, ^57^ Previous work from Zheng and coworkers ^27^ showed that 2% O_2_ improved ROS generation via nuclear NADPH oxidase 4, which was direct correlated with the increase of hair inductivity of human DP cells. Thus, the oxygen levels in the highly compact DP‐hMel aggregates are also likely to drop below 5% O_2_ and lead to the formation of ROS by an alternative mechanism, other than the one occurring in the indirect co‐cultures.^24^ Moreover, ROS increase in the 3D‐cellular aggregates may also be a consequence of the improved tyrosinase activity which is, by itself, a ROS‐generating oxidative step.^17^, ^58^ Moreover, albeit a direct correlation was not observed in flat cultures, in the 3D‐cell aggregates both ROS levels and ALP activity were higher in physoxia than in normoxia, demonstrating that in a more complex environment the interactions between hMel and DP cells can be better represented.

## 5 CONCLUSIONS

In summary, our results demonstrate that the recreation of the HF oxygen levels and the associated decrease of intracellular ROS benefit both hMel and DP cells, improving their proliferative capacity and functional features. Furthermore, we show that the type of interaction occurring between these cells also affects their response to physoxia and that, within the 3D‐aggregates, both hMel and DP cell‐type functions and ROS generation are increased. Taken together, our results demonstrate that hMel‐DP cells direct interaction under physiological oxygen levels has a superior capacity to recreate a microenvironment with features representative of the anagen bulb milieu, consequently enhancing their cell‐specific functions.

## CONFLICT OF INTEREST

The authors declare that they have no competing interests.

## AUTHOR CONTRIBUTIONS

CA: conceptualization, investigation, methodology, data curation, formal analysis, visualization, writing—original draft preparation; APM: funding acquisition, project administration, resources, conceptualization, supervision, validation, writing—review and editing; RLR: resources, funding acquisition.

## Supporting information

Supplementary Material

## Data Availability

The datasets used and/or analysed during the current study are available from the corresponding author on reasonable request.
