# Supplementary material for: Dermal papilla cells and melanocytes response to physiological oxygen levels depends on their interactions
Source: Cell Prolif. 2021 Jun 8;54(7):e13013. doi: 10.1111/cpr.13013 (PMC8249782; doi:10.1111/cpr.13013)
Supplement: Supplementary file 1 — Supplementary Material [file CPR-54-e13013-s001.docx]

**Supplementary information**

**Dermal papilla cells and melanocytes response to physiological oxygen levels depends on their interactions**

Carla M. Abreu^1,2^, Rui L. Reis^1,2^, Alexandra P. Marques^1,2*^

^1^ 3B’s Research Group – Biomaterials, Biodegradables and Biomimetics, University of Minho, Avepark 4805-017 Barco, Guimarães, Portugal

^2^ ICVS/3B’s – PT Government Associate Laboratory, Braga/Guimarães, Portugal

**Supplementary Table**

**Table S1 -** List of the antibodies used for immunofluorescence studies

| **Antibody** | **Supplier** | **Host species** | **Type** | **Dilution** | **Reference** |
| --- | --- | --- | --- | --- | --- |
| Tyrosinase | SCBT | Mouse | Monoclonal | 1:100 | sc-20035 |
| Vimentin | Abcam | Rabbit | Monoclonal | 1:50 | ab92547 |
| PMEL | Thermo Fisher Scientific | Mouse | Monoclonal | 1:100 | MA1-34759 |
| S100 | Dako | Rabbit | Polyclonal | 1:100 | Z031129-2 |
| Versican V2 | DSHB | Mouse | Monoclonal | 1:10 | 12C5 |
| MelanA | Abcam | Rabbit | Monoclonal | 1:250 | ab51061 |
| Ki67 | Abcam | Rabbit | Monoclonal | 1:50 | ab16667 |

SCBT: Santa Cruz Biotechnology; DSHB: [Developmental Studies Hybridoma Bank](http://dshb.biology.uiowa.edu/)

**Supplementary Figure**


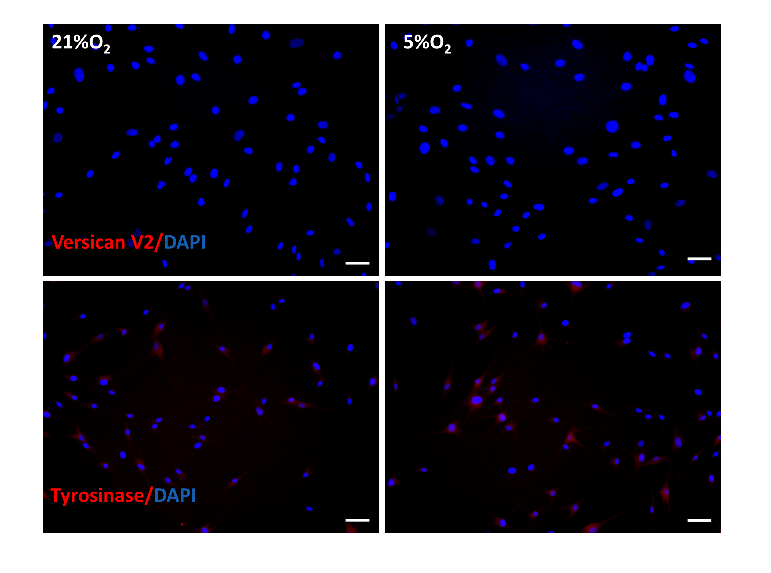


**Fig. S1.** Representative immunofluorescence images showing lack of expression of the V2-isoform of versican by DP cells (upper panel) and the low tyrosinase expression by hMel (lower panel) in 2D-cultures under normoxia and physoxia. Nuclei were counterstained with DAPI. Scale bars = 50 μm.
